# Fission yeast mtr1p regulates interphase microtubule cortical dwell-time

**DOI:** 10.1242/bio.20148607

**Published:** 2014-06-13

**Authors:** Frédérique Carlier-Grynkorn, Liang Ji, Vincent Fraisier, Berangère Lombard, Florent Dingli, Damarys Loew, Anne Paoletti, Xavier Ronot, Phong T. Tran

**Affiliations:** 1 Institut Curie, Paris 75005, France; 2 CNRS, UMR 144, Paris 75005, France; 3 Laboratoire CaCyS, FRE AGIM 3405 UJF-CNRS-EPHE-UMPF, La Tronche 38700, France; 4 Department of Cell and Developmental Biology, University of Pennsylvania, Philadelphia, PA 19104, USA

**Keywords:** Dynamic instability, Microtubule, Ribosome

## Abstract

The microtubule cytoskeleton plays important roles in cell polarity, motility and division. Microtubules inherently undergo dynamic instability, stochastically switching between phases of growth and shrinkage. In cells, some microtubule-associated proteins (MAPs) and molecular motors can further modulate microtubule dynamics. We present here the fission yeast *mtr1^+^*, a new regulator of microtubule dynamics that appears to be not a MAP or a motor. *mtr1*-deletion (mtr1Δ) primarily results in longer microtubule dwell-time at the cell tip cortex, suggesting that mtr1p acts directly or indirectly as a destabilizer of microtubules. mtr1p is antagonistic to mal3p, the ortholog of mammalian EB1, which stabilizes microtubules. mal3Δ results in short microtubules, but can be partially rescued by mtr1Δ, as the double mutant mal3Δ mtr1Δ exhibits longer microtubules than mal3Δ single mutant. By sequence homology, mtr1p is predicted to be a component of the ribosomal quality control complex. Intriguingly, deletion of a predicted ribosomal gene, *rps1801*, also resulted in longer microtubule dwell-time similar to mtr1Δ. The double-mutant mal3Δ rps1801Δ also exhibits longer microtubules than mal3Δ single mutant alone. Our study suggests a possible involvement of mtr1p and the ribosome complex in modulating microtubule dynamics.

## INTRODUCTION

The microtubule cytoskeleton plays essential roles in many cellular processes such as cell polarity, motility and division (Mimori-Kiyosue, 2011). Microtubules are polar polymers composed of αβ-tubulin heterodimers and are inherently dynamic, frequently switching between phases of growth and shrinkage (Mitchison and Kirschner, 1984). In the cell, microtubule dynamics are further regulated by various microtubule-associated proteins (MAPs) and molecular motors (Mimori-Kiyosue, 2011). In general, microtubule dynamics can be regulated at the minus ends, the microtubule lattice, or the plus ends. At the minus ends, the microtubule-organizing center (MTOC) and the gamma-tubulin complex (γ-TuRC) control the nucleation of microtubules (Kollman et al., 2011; Teixidó-Travesa et al., 2012), which dictates the number of microtubules throughout the cell cycle (Sawin and Tran, 2006). At the microtubule lattice, neuronal MAPs such as Tau and MAP2 can modulate microtubule dynamics (Matenia and Mandelkow, 2009), although their major roles may be to fortify the microtubule lattice stiffness (Felgner et al., 1997). At the plus ends, a class of MAPs termed the plus end tip tracking proteins (+TIPs) (Galjart, 2010) constitute the key players of the microtubule dynamics characterized by four parameters – velocities of growth and shrinkage, and frequencies of catastrophe and rescue, V_g_, V_s_, F_cat_, F_res_, respectively (Walker et al., 1988).

Of the +TIP proteins, EB1 (mal3p in fission yeast) appears to have major roles in controlling microtubule dynamics. EB1 itself stabilizes microtubules by suppressing catastrophe (Busch and Brunner, 2004) or suppressing shrinkage (Katsuki et al., 2009). Further, EB1 also recruits many other +TIP proteins, which can themselves regulate microtubule dynamics (Busch and Brunner, 2004). A major determinant for EB1-dependent recruitment to the microtubule plus end is characterized by the existence of a disordered region containing the motif SxIP and flanking basic amino acids (Honnappa et al., 2009). Indeed, this motif has been highly successful in predicting novel +TIP proteins in mammalian cells (Jiang et al., 2012).

Here, we used the SxIP motif to identify new fission yeast proteins able to regulate microtubule dynamics. The fission yeast *Schizosaccharomyces pombe* has been a good model system to analyze microtubules and their diverse functions in cells (Martin, 2009; Piel and Tran, 2009). Our screen reveals *mtr1^+^* as a new fission yeast regulator of interphase microtubule dynamics. mtr1p appears to increase the frequency of catastrophe, a role antagonistic to mal3p. Surprisingly, mtr1p does not localize to the microtubule plus end, as mal3p does. Intriguingly, mtr1p, as the ortholog of *S. cerevisiae* TAE2, is predicted to be a component of the ribosome quality control complex. We show that a predicted component of the ribosome, the S18 complex protein rps1801p, which interacts genetically with mtr1p ortholog in budding yeast, acts similar to mtr1p in destabilizing microtubules.

It has been reported that ribosomes can localize to microtubules (Hamill et al., 1994; Suprenant et al., 1989), presumably to facilitate the transport of certain mRNAs to specific cellular compartments (Beach et al., 1999; Bertrand et al., 1998). Our results suggest a possible new function for ribosomes, that of regulating microtubule dynamics in a direct or indirect manner.

## MATERIALS AND METHODS

### *S. pombe* strains and plasmids construction

Standard yeast media and genetic methods were used to create yeast strains, as previously described (Forsburg and Rhind, 2006; Moreno et al., 1991). Strains of deletion and GFP/mCherry tagging were carried out by the PCR-based method previously described (Bähler et al., 1998). All strains used in this study are listed in supplementary material Table S1.

### Bioinformatic screen for *mtr1^+^*

We utilized the *S. pombe* haploid deletion collection (Kim et al., 2010) (http://www.bioneer.com) to identify novel genes whose deletion lead to microtubule-based defects. Uncharacterized genes containing the SxIP motif, a predictor of mal3p/EB1 binding (Honnappa et al., 2009), were examined. The novel gene *SPCC132.01c* was found to have interphase microtubule defects. We thus named this gene *mtr1^+^* (microtubule regulator 1).

### Microscopy

Live cell imaging was carried out at room temperature 25°C. We use a spinning-disc confocal microscope equipped with a Nikon PlanApo 100×/1.40 NA objective and the Photometrics CoolSNAP HQ2 CCD camera, as previously described (Tran et al., 2004). MetaMorph 7.5 (http://www.moleculardevices.com) was used to acquire and process all images.

For high temporal resolution, images were acquired at 300–500 ms exposure for GFP/mCherry, 5-sec intervals, 10 min total time for two optical sections of 0.3 µm spacing. For longer time scale, images were acquired at 300–500 ms exposures for GFP/mCherry, 30-sec intervals, with each stack comprising 11 optical sections of 0.5 µm spacing.

We note that in our hands, tubulin tagged with GFP resulted in slightly different microtubule dynamics than tubulin tagged with mCherry. For example, wild-type microtubule dwell-time was higher when measured with GFP-atb2 compared to mCherry-atb2. For this reason, comparisons of microtubule dynamic parameters between wild-type and mutant strains were strictly performed on strains expressing the same tagged tubulin.

### Data analysis

Data are presented as mean ± s.d. Statistical analysis on means were performed using the Student t-test and statistical analysis on frequencies were performed using the Chi-squared test, in Microsoft Office Excel 2010. All plots were created using Kaleidagraph 4.0 (http://www.synergy.com). Box plots show all individual data points, and the plots enclose 50% of the data in the box with the median value displayed as a line. The lines extending from the top and bottom of each box mark the minimum and maximum values within the data set that fall within an acceptable range. Outliers are displayed as individual points.

## RESULTS

In a bioinformatic screen for new fission yeast proteins containing the SxIP motif predicted to bind to EB1/mal3p (Honnappa et al., 2009), we identified the previously uncharacterized gene *SPCC132.01c*. Deletion of this gene resulted in interphase microtubule phenotypes (see below). Thus we named this gene *mtr1^+^* (microtubule regulator 1).

### mtr1p decreases interphase microtubule dwell-time and increases the frequency of catastrophe

**Fig. 1.**
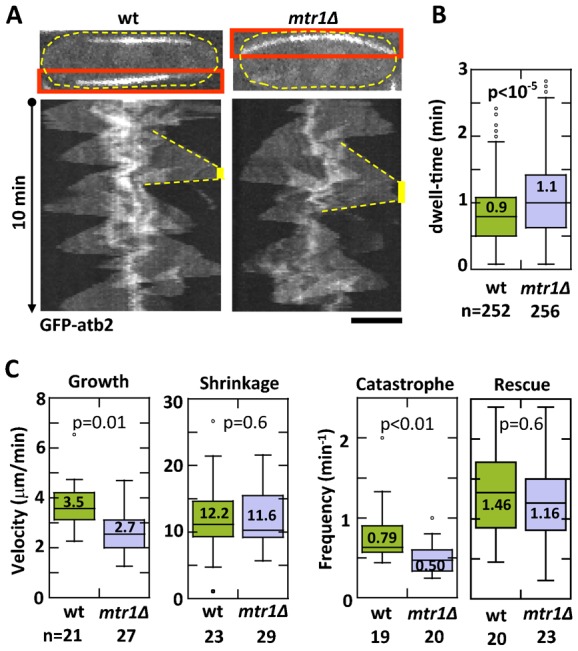
mtr1p modulates interphase microtubule dynamics. (A) Kymographs of wild-type and mtr1Δ cells expressing GFP-atb2 (tubulin). Red box shows the individual interphase microtubule used in the kymograph. Kymograph shows microtubule dynamics for 10 min total time. Several cycles of growth and shrinkage are evident. Yellow bar indicates the dwell-time, defined as the duration where the microtubule plus tip stayed in contact with the cell tip cortex. (B) Box plot comparison of interphase microtubule dwell-time of wild-type and mtr1Δ cells expressing GFP-atb2. Wild-type cells show a dwell-time of 0.9±0.4 min (n = 256). In contrast, mtr1Δ cells showed a dwell-time of 1.1±0.6 min (n = 252), an ∼20% prolongation of dwell-time (p<10^−5^). (C) Box plot comparison of interphase microtubule dynamic instability parameters – velocities of growth (V_g_) and shrinkage (V_s_), and frequencies of catastrophe (F_cat_) and rescue (F_res_) – of wild-type and mtr1Δ cells expressing GFP-atb2. For wild-type cells, V_g_ was 3.5±1.2 µm/min (n = 21), V_s_ was 12.2±5.6 µm/min (n = 23), F_cat_ was 0.79±0.38 per min (n = 19), and F_res_ was 1.46±0.70 per min (n = 20). For mtr1Δ cells, V_g_ was 2.7±0.9 µm/min (n = 27), V_s_ was 11.6±4.0 µm/min (n = 29), F_cat_ was 0.50±0.20 per min (n = 20), and F_res_ was 1.16±0.59 per min (n = 23). Effectively, V_g_ for mtr1Δ was ∼23% less than wild type (p<0.02), V_s_ for mtr1Δ was similar to wild type (p = 0.65), F_cat_ for mtr1Δ was ∼37% less than wild type (p<0.01), and F_res_ for mtr1Δ was similar to wild type (p = 0.14). Scale bar: 5 µm.

**Fig. 2.**
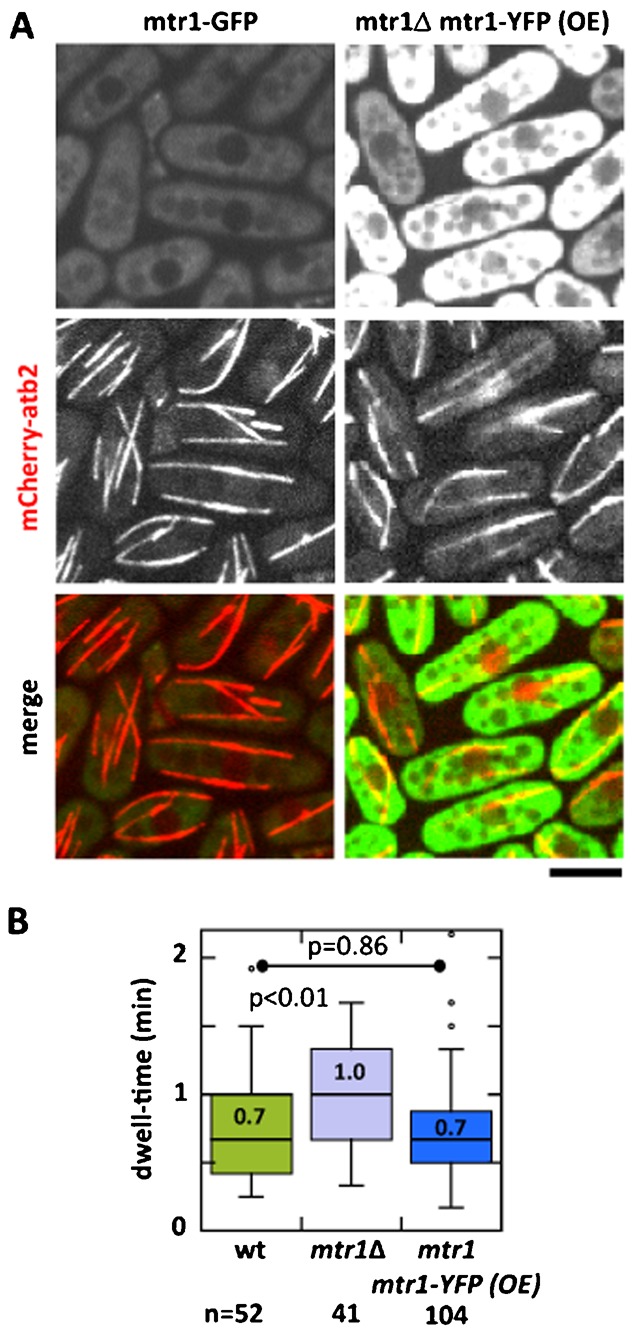
mtr1p is cytoplasmic. (A) Maximum-projection images of wild-type cells expressing endogenous level of mtr1-GFP (mtr1 promoter) compare with mtr1Δ cells over-expressing mtr1-YFP (thiamine-repressible nmt1 promoter). All cells also express mCherry-atb2. Induced over-expression for 16 hrs indicates an approximately 3-fold signal intensity increase. mtr1p signal is uniformly in the cytoplasm and is not observed in the nucleus and the vacuoles. (B) Box plot comparison of interphase microtubule dwell-time of wild-type mtr1-GFP, mtr1Δ, and mtr1Δ mtr1-YFP (over-expressed) cells. Cells also expressed mCherry-atb2. Whereas the wild type showed a dwell-time of 0.7±0.4 min (n = 52), and mtr1Δ showed an ∼30% increase (p<0.01) to 1.0±0.4 min (n = 42), the over-expressed mtr1Δ mtr1-YFP cells showed a similar dwell-time (p = 0.86) to wild type at 0.7±0.5 min (n = 104). This indicates that ectopic expression of mtr1-YFP in mtr1Δ cells rescued the dwell-time defects, and presumably other microtubule dynamic defects, of mtr1Δ. Scale bar: 5 µm.

We deleted *mtr1^+^*, and then compared microtubule dynamics in wild-type and mtr1Δ cells expressing fluorescent GFP-atb2p (tubulin). In general, microtubule defects in mtr1Δ cells were very subtle, with no measurable defects in spindle formation or elongation (data not shown). We did observe subtle defects in interphase microtubule dynamics in mtr1Δ cells. While the typical 3–4 bundles of interphase microtubules were the same for both wild-type and mtr1Δ cells (data not shown), parameters of individual microtubule dynamics differed. The most consistently noticeable behavior of the interphase microtubules is their dwell-time at the cell tips (Fig. 1A). Dwell-time is defined as the contact time between the growing microtubule and the cell cortex (Tran et al., 2001). Wild-type cells showed a dwell-time of 0.9±0.4 min (n = 256). In contrast, mtr1Δ cells showed a dwell-time of 1.1±0.6 min (n = 252), an ∼20% prolongation of dwell-time (p<10^−5^) (Fig. 2B). This result immediately suggests that the protein mtr1p functions to decrease microtubule dwell-time. In other words, mtr1p decreases microtubule stability.

Microtubule stability is defined by the four parameters of microtubule dynamics – V_g_, V_s_, F_cat_, and F_res_ (Walker et al., 1988). To understand which parameters are modulated by mtr1p, we measured and compared these four parameters in wild-type and mtr1Δ cells expressing GFP-atb2p. For wild-type cells, V_g_ was 3.5±1.2 µm/min (n = 21), V_s_ was 12.2±5.6 µm/min (n = 23), F_cat_ was 0.79±0.38 per min (n = 19), and F_res_ 1.46±0.70 per min (n = 20) (Fig. 1C). For mtr1Δ cells, V_g_ was 2.7±0.9 µm/min (n = 27), V_s_ was 11.6±4.0 µm/min (n = 29), F_cat_ was 0.50±0.20 per min (n = 20), and F_res_ 1.16±0.59 per min (n = 23) (Fig. 1C). Effectively, V_g_ for mtr1Δ was ∼23% less than wild type (p<0.02), V_s_ for mtr1Δ was similar to wild type (p = 0.65), F_cat_ for mtr1Δ was ∼37% less than wild type (p<0.01), and F_res_ for mtr1Δ was similar to wild type (p = 0.14). This suggests that mtr1p plays no significant role in modulating microtubule shrinkage velocity or the frequency of rescue. However, mtr1p appears to help increase microtubule growth velocity and to increase the frequency of catastrophe. Further, the role of mtr1p in increasing the frequency of catastrophe is consistent with its role in decreasing the microtubule dwell-time.

### mtr1p fluorescent signal is strictly cytoplasmic

The fission yeast +TIP protein mal3p (mammalian EB1) localizes to the growing interphase microtubule plus ends (Beinhauer et al., 1997; Busch and Brunner, 2004). As mtr1p contains the SxIP motif predicted to bind to mal3p (Honnappa et al., 2009), and as mtr1p functions in modulating microtubule dynamics (Fig. 1), we reasoned that mtr1p may be a +TIP protein. We thus tagged GFP at the C-terminus of the *mtr1^+^* locus, and observed mtr1-GFP localization with respect to microtubules (mCherry-atb2). Surprisingly, endogenous-level expression of mtr1-GFP showed that mtr1p is cytoplasmic, and excluded from the nucleus and vacuoles (Fig. 2A). No co-localization of mtr1p with microtubules was observed with our current imaging setup (Fig. 2A). We next over-expressed mtr1-YFP, using the thiamine-repressible nmt1 promoter ectopically expressed at the *leu1^+^* locus (Maundrell, 1993). Again, at very high mtr1-YFP expression level, as judged by the comparatively high fluorescent signal of 3-fold increase, we only observed mtr1p uniformly in the cytoplasm (Fig. 2A).

To confirm that the over-expressed mtr1-YFP was functional, we tested if the over-expressed mtr1p can rescue the microtubule defects present in mtr1Δ cells. Specifically, we compared the interphase microtubule dwell-time of wild-type, mtr1Δ, and mtr1Δ mtr1-YFP(OE) cells expressing mCherry-atb2. Ectopic over-expression of mtr1-YFP indeed rescued the prolonged dwell-time of mtr1Δ (Fig. 2B). In these experiments, whereas the wild type showed a dwell-time of 0.7±0.4 min (n = 52), and mtr1Δ showed an ∼30% increase to 1.0±0.4 min (n = 42, p<0.01), the over-expressed mtr1Δ mtr1-YFP(OE) cells showed a similar dwell-time to wild type at 0.7±0.5 min (n = 104, p = 0.86). Thus, ectopically expressing mtr1-YFP can rescue the mtr1Δ deletion, suggesting that mtr1-YFP tag was functional and behaved like wild-type mtr1p. Interestingly, over-expression of mtr1-YFP did not enhance its function, meaning over-expression did not decrease the microtubule dwell-time compared to wild type. This suggested that mtr1p function is not concentration-dependent.

There may be the possibility that weak and/or transient putative interaction of mtr1p with the microtubule plus end prevented its detection with our current imaging setup. Nevertheless, the endogenous and over-expression results suggest that mtr1p is not a +TIP protein, but does somehow modulate microtubule dynamics.

### mtr1p is antagonistic to mal3p

**Fig. 3.**
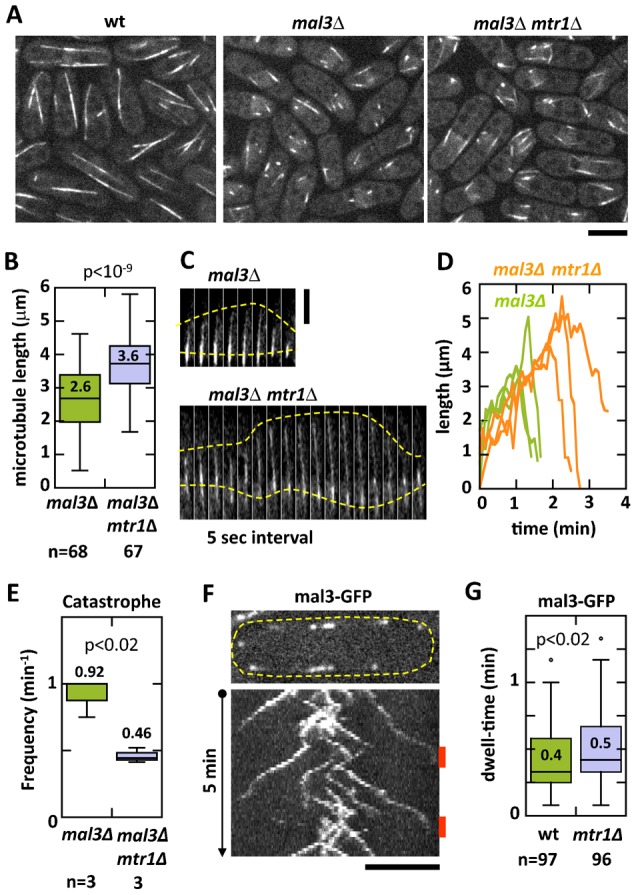
mtr1p is antagonistic to mal3p. (A) Maximum-projection images of wild-type, mal3Δ, and mal3Δ mtr1Δ cells expressing GFP-atb2. Wild-type cells have relatively long interphase microtubules. In contrast, mal3Δ cells have very short microtubules; and the mal3Δ mtr1Δ double-deletion cells also have short microtubules. (B) Box plot comparison of interphase microtubule lengths of mal3Δ and mal3Δ mtr1Δ cells expressing GFP-atb2. At any given time point, cells will contain microtubules of different lengths, many too short to be confidently and reliably measured. We chose to measure the longest interphase microtubule bundle in each cell of the population. The mal3Δ cells had an average interphase microtubule length of 2.6±0.9 µm (n = 68). In contrast, the mal3Δ mtr1Δ cells had longer microtubule length of 3.6±0.8 µm (n = 67), an ∼40% increase in length (p<10^−9^). (C) Time-lapse images of longest interphase microtubule dynamics of mal3Δ and mal3Δ mtr1Δ cells expressing GFP-atb2. The longest interphase microtubules of mal3Δ mtr1Δ cells are consistently longer than the longest interphase microtubules of mal3Δ cells. (D) Plot of individual longest interphase microtubule dynamics of mal3Δ and mal3Δ mtr1Δ cells expressing GFP-atb2. The velocities of growth and shrinkage of the microtubules appear qualitatively the same, as judged by the slopes. The mal3Δ mtr1Δ microtubules consistently grow longer and undergo catastrophe less frequently than mal3Δ microtubules. (E) Box plot comparison of the frequency of catastrophe F_cat_ between the mal3Δ and mal3Δ mtr1Δ microtubules shown in panel D. For mal3Δ, the F_cat_ was 0.92±0.14 per min (n = 3). In contrast, formal3Δ mtr1Δ, the F_cat_ was 0.46±0.06 per min (n = 3), an ∼50% decrease (p<0.02). (F) Kymograph of wild-type cell expressing mal3-GFP. mal3p is a microtubule plus end tracking protein. Kymograph shows mal3-GFP dynamics for 5 min total time. Two cycles of growth and shrinkage are evident. Orange bar indicates the dwell-time, defined as the duration where mal3-GFP stayed in contact with the cell tip cortex. (G) Box plot comparison of mal3p dwell-time of wild-type and mtr1Δ cells expressing mal3-GFP. Whereas wild-type mal3-GFP dwell-time was 0.4±0.2 min (n = 97), the mtr1Δ mal3-GFP dwell-time was 0.5±0.2 min (n = 96), an ∼25% increase (p<0.02). This is consistent with the ∼20% prolonged interphase microtubule dwell-time found in mtr1Δ in Fig. 1B. Scale bars: 5 µm (A,F), 1 µm (C).

The results thus far suggest that mtr1p destabilized interphase microtubules in fission yeast. The fission yeast mal3p has been shown to stabilize microtubules (Beinhauer et al., 1997; Busch and Brunner, 2004), consistent with its role as the EB1 +TIP protein. This suggests that mtr1p and mal3p may be antagonistic. We tested the hypothesis that by removing this antagonism, for example in the double-deletion strain mal3Δ mtr1Δ, we can rescue the microtubule defects contributed by individual deletion of mal3Δ or mtr1Δ. We compared wild-type, mal3Δ, and mal3Δ mtr1Δ strains expressing GFP-atb2. Qualitatively, wild-type cells showed long bundles of interphase microtubules that can reach the cell tips (Fig. 3A). In contrast, mal3Δ cells showed very short interphase microtubule bundles (Fig. 3A), consistent with previous reports (Beinhauer et al., 1997; Busch and Brunner, 2004). The mal3Δ mtr1Δ cells also showed short interphase microtubule bundles, but which appeared slightly longer than in mal3Δ alone (Fig. 3A).

We measured and compared the length of individual interphase microtubules in mal3Δ and mal3Δ mtr1Δ cells expressing GFP-atb2 (Fig. 3B). As there were many very short microtubules, we limited our analysis to the longest microtubule bundle observed in each cell. This limitation did not take into account the short microtubules, which were difficult to measure with high precision. Based upon this definition, the mal3Δ cells had an average interphase microtubule length of 2.6±0.9 µm (n = 68). In contrast, the mal3Δ mtr1Δcells had a longer microtubule length of 3.6±0.8 µm (n = 67), an ∼40% increase in length (p<10^−9^). This result is consistent with mtr1p having a destabilizing effect on microtubules. For additional confirmation, we measured and compared individual interphase microtubule dynamics, focusing on the few longest microtubules of both mal3Δ and mal3Δ mtr1Δ cells expressing GFP-atb2 (Fig. 3C). Qualitatively, both V_g_ and V_s_ appeared similar for both mal3Δ and mal3Δ mtr1Δ cells (Fig. 3D). The most prominent difference was that microtubules in mal3Δ mtr1Δ cells grew longer and for longer time duration compared to mal3Δ alone (Fig. 3D). This resulted in a decrease in the frequency of catastrophe for mal3Δ mtr1Δ cells. For mal3Δ, the F_cat_ was 0.92±0.14 per min (n = 3), which was similar to published reports (Busch and Brunner, 2004). In contrast, for mal3Δ mtr1Δ, the F_cat_ was 0.46±0.06 per min (n = 3), an ∼50% decrease (p<0.02) (Fig. 3E). Our results are consistent with mtr1p and mal3p playing antagonistic roles in microtubule dynamics regulation.

We did not observe mtr1p at microtubule plus ends (Fig. 2). Nevertheless, given mtr1p antagonistic function to mal3p, we examined mal3p dynamics in wild-type and mtr1Δ cells. Strains simultaneously expressing mal3-GFP and mCherry-atb2 (or the inverse) were not functional, exhibiting abnormally short microtubules (data not shown). We therefore imaged mal3-GFP in wild-type and mtr1Δ cells (Fig. 3F). We did not observe significant differences in mal3-GFP intensities at the microtubule plus ends between wild-type and mtr1Δ cells (data not shown). However, the dwell-time of mal3-GFP signal at the cell tip cortex was different between wild-type and mrt1Δ cells (Fig. 3G). Whereas wild-type mal3-GFP dwell-time was 0.4±0.2 min (n = 97), the mtr1Δ mal3-GFP dwell-time was 0.5±0.2 min (n = 96), an ∼25% increase (p<0.02). It is reported that microtubule catastrophe and shrinkage follow the unbinding of mal3p from the microtubule plus end (Maurer et al., 2012). Thus, that mtr1Δ showed longer mal3p dwell-time (Fig. 3G) is consistent with the result that mtr1Δ led to longer microtubule dwell-time (Fig. 1B), i.e. longer mal3p dwell-time promotes longer microtubule growth and less microtubule catastrophe. Further, that the double-deletion mal3Δ mtr1Δ showed longer microtubules than mal3Δ alone suggests that mal3p and mtr1p act independently of each other when antagonistically regulating microtubule dynamics.

### mtr1p is evolutionarily conserved

**Fig. 4.**
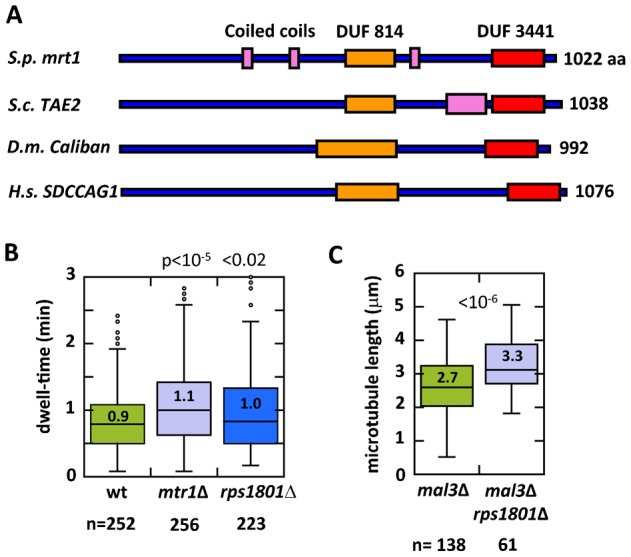
mtr1p is evolutionarily conserved. (A) Predicted domain structure of mtr1p. DUF (orange, red), domain of unknown function; Coiled coils (pink). *S. pombe* mtr1 has orthologs in *S. cerevesaie* TAE2, *D. melanogaster* Caliban, and *H. sapiens* SDCCAG1. See supplementary material Fig. S1 for the complete amino acid alignment. (B) Box plot comparison of interphase microtubule dwell-time of wild-type, mtr1Δ, and predicted ribosome complex gene rps1801Δ cells expressing GFP-atb2. Whereas wild-type microtubule dwell-time was 0.9±0.5 min (n = 252), mtr1Δ was longer at 1.1±0.6 min (n = 256) (p<10^−5^), and rps1801Δ was longer at 1.0±0.6 min (n = 223) (p<0.02). (C) Box plot comparison of interphase microtubule lengths of mal3Δ and mal3Δ rps1801Δ cells expressing GFP-atb2. At any given time point, cells will contain microtubules of different lengths, many too short to be precisely measured. We chose to measure the longest interphase microtubule bundle in each cell of the population. The mal3Δ cells had an average interphase microtubule length of 2.7±0.9 µm (n = 138). In contrast, the mal3Δ rps1801Δ cells had longer microtubule length of 3.3±0.8 µm (n = 61), an ∼20% increase in length (p<10^−6^).

BLAST analysis revealed that mtr1p is evolutionarily conserved, with orthologs (33–37% identical amino acid, and E-value of 10^−102^–10^−137^) from yeast to human (Fig. 4A; supplementary material Fig. S1). The human ortholog SDCCAG1 (NEMF) is implicated in colon and lung cancer (Carbonnelle et al., 1999), the *Drosophila* ortholog *Caliban* is reported to be a tumor suppressor gene and mediates protein nuclear export (Bi et al., 2005), and the budding yeast TAE2 (translation-associated element 2) is associated with the ribosome quality control complex and regulates degradation of defective translation products (Brandman et al., 2012; Defenouillère et al., 2013). We currently have no direct evidence that mtr1p is part of an evolutionarily conserved ribosome quality control complex in fission yeast, or that mtr1p can associate with ribosomes. Nevertheless, we tested whether the fission yeast ortholog of another budding yeast gene encoding a component of the ribosomal complex and showing synthetic genetic interaction with TAE2, also exhibited defects in microtubule dynamics (Alamgir et al., 2010). The previously uncharacterized fission yeast gene *rps1801^+^* (SPBC16D10.11c) is currently predicted to encode a ribosome complex protein (http://www.pombase.org). We compared the microtubule dwell-time among wild-type, mtr1Δ, and rps1801Δ cells expressing GFP-atb2 (Fig. 4B). Wild-type microtubule dwell-time was 0.9±0.5 min (n = 252), mtr1Δ was longer at 1.1±0.6 min (n = 256) (p<10^−5^), and rps1801Δ was longer at 1.0±0.6 min (n = 223) (p<0.02). This suggests that in addition to mtr1p, rps1801p has similar microtubule destabilizing effects. We further tested if rps1801Δ was also antagonistic to mal3Δ. mal3Δ cells had an average interphase microtubule length of 2.7±0.8 µm (n = 138). In contrast, the mal3Δ rps1801Δ cells had a longer microtubule length of 3.3±0.8 µm (n = 61), an ∼20% increase in length (p<10^−6^) (Fig. 4C). The results suggest that mtr1p and at least one other predicted ribosome complex protein, rps1801p, have direct or indirect function in modulating microtubule dynamics.

## DISCUSSION

Our present study implicates mtr1p, a putative ribosome complex protein, and one other putative ribosome complex protein, rps1801p, in modulating microtubule dynamics. mtr1-deletion leads to subtle but significant stabilization of interphase microtubules, particularly through prolongation of dwell-time of microtubule plus ends at the cell tip cortex, and the decrease in the frequency of catastrophe (Fig. 1). While mtr1p was discovered from a bioinformatic screen for +TIP mal3p-interacting proteins, it surprisingly does not localize to the microtubule plus end like mal3p does. Instead, mtr1p is cytoplasmic (Fig. 2). This implies that we serendipitously discovered mtr1p, and that its SxIP domain has no role in mal3p binding. Interestingly, mtr1p and mal3p are antagonistic – mtr1p destabilizes microtubules and mal3p stabilizes microtubules (Fig. 3). That mtr1p and mal3p do not share co-localization at the microtubule plus tips, and that the double-deletion mal3Δ mtr1Δ partially rescued the short microtubule phenotypes of mal3Δ alone, suggest that mtr1p and mal3p act in distinct pathways regulating microtubule dynamics. mtr1p is predicted to be a ribosome complex protein. We found that one other predicted ribosome complex protein, rps1801p, also acts similarly to mtr1p in regulating microtubule dynamics (Fig. 4).

*mtr1^+^* was initially found from a bioinformatic screen for potential mal3p/EB1 binding proteins containing the SxIP motif. Our results suggest that mtr1p is not localized at the microtubule plus tip, as mal3p does. Further, we also failed to detect mal3p in a mass-spectrometry study using mtr1-GFP and GFP control pull downs (data not shown). Thus, there is no detectable interaction between mtr1p and mal3p. In this instance, the SxIP domain was not a good predictor of mal3p binding. That mtr1p has a role in microtubule dynamics was serendipitous.

The effect of mtr1-deletion on microtubule stability is relatively minor, with slight increase in dwell-time and slight decrease in the frequency of catastrophe. We failed to observe down stream consequences of mtr1Δ, such as cell shape and cell length defects or mitotic defects associated with microtubule defects. Nevertheless, mtr1p destabilizing effect on microtubules appears bona fide, as mtr1p is antagonistic to mal3p. At the moment, we are far from understanding how mtr1p regulates microtubule dynamics. Its cytoplasmic distribution offers no clues to its mechanism of action.

Fission yeast mtr1p is predicted to be a ribosome complex protein based on its sequence homology to the budding yeast ribosome complex-interacting protein TAE2 (Brandman et al., 2012; Defenouillère et al., 2013). TAE2 is implicated in a ribosomal quality control pathway to degrade aberrant translational products (Brandman et al., 2012; Defenouillère et al., 2013). While we currently have no definitive proof that mtr1p is a ribosomal protein, mass-spectrometry data of mtr1-GFP pull-down, compared to data of its control GFP pull-down, appear slightly enriched in ribosomal proteins (data not shown). The mtr1-GFP pull down showed an enrichment factor of 1.37× (p<10^−10^) in ribosomal proteins compared to the control GFP pull down. In addition, coupled with the fact that one other fission yeast predicted ribosome complex protein, rps1801p, also showed similar microtubule dynamic phenotypes to mtr1p, suggest that the ribosome complex may function in regulating microtubule dynamics. Perhaps, as part of the ribosome quality control complex degrading aberrant translational products, mtr1p ensures that some microtubule regulatory proteins are properly translated.

We failed to observe enhanced cycloheximide sensitivity, based on colony growth assay, in mtr1Δ cells compared to wild type (data not shown). Therefore, we do not favor, but cannot rule out, the model that mtr1p may affect global protein translation, including translation of microtubule regulatory proteins, which would subsequently modify microtubule dynamics. Intriguingly, there is old evidence that the ribosome complex proteins can bind directly to the microtubule lattice (Suprenant et al., 1989); and there is also evidence that the microtubule-associated protein EMAP can bind to ribosomes (Suprenant et al., 1993). While we show no evidence that mtr1p binds either microtubules or ribosomes directly, we can imagine scenarios where mtr1p, as part of the ribosome-microtubule interactor, slightly destabilizes the microtubule lattice by virtue of its binding. Alternatively, as a primarily cytoplasmic protein, mtr1p may transiently interact with the microtubule plus tips to regulate dynamics.

mtr1p is evolutionarily conserved (Fig. 4). Interestingly, the *Drosophila* and human orthologs *Caliban* and SDCCAG1, respectively, have been shown to be mediators of nuclear export (Bi et al., 2005). Further, *Caliban* has been proposed to be a tumor suppressor. Lung carcinomas and colon cancer cells have nonfunctional SDCCAG1 (Carbonnelle et al., 1999; Scanlan et al., 1998). Ectopic expression of *Caliban* in human lung carcinomas reduced their proliferation and invasiveness (Bi et al., 2005). As cell motility and mitosis are performed by the microtubule cytoskeleton, it is tempting to propose that SDCCAG1, like mtr1p, may regulate microtubule dynamics.

## Supplementary Material

Supplementary Material
