# Supplementary material for: Fission yeast mtr1p regulates interphase microtubule cortical dwell-time
Source: Biol Open. 2014 Jun 13;3(7):591–6. doi: 10.1242/bio.20148607 (PMC4154295; doi:10.1242/bio.20148607)
Supplement: Supplementary Material [file supp_3_7_591__index.html]

Fission yeast mtr1p regulates interphase microtubule cortical dwell-time — Supplementary Material 

# Fission yeast mtr1p regulates interphase microtubule cortical dwell-time

## bio.20148607 Supplementary Material

**Files in this Data Supplement:**

- Supplementary Material - Frédérique Carlier-Grynkorn et al. doi: 10.1242/bio.20148607
