# Supplementary material for: Fission yeast mtr1p regulates interphase microtubule cortical dwell-time
Source: Biol Open. 2014 Jun 13;3(7):591–6. doi: 10.1242/bio.20148607 (PMC4154295; doi:10.1242/bio.20148607)
Supplement: Supplementary Material [file supp_bio.20148607_bio.20148607-s1.pdf]

Biology Open (2014) 000, 1–6 doi:10.1242/bio.20148607

Supplementary Material

Frédérique Carlier-Grynkorn et al. doi: 10.1242/bio.20148607

```
Sp mtr1               MKQRFSALDIAAIAAELREQVVGCRLNNFYDLNARTFLLKFG--KQDAKYSIVIESGFRA 58
Sc TAE2               MKQRISALDLLLLARELKQDLEGYRLSNIYNIADSSKQFLLKFNKPDSKLNVVVDCGLRI 60
Dm Caliban            MKTRFNTFDIICGVAELQK-LVGWRVNQIYDVDNKTYLFRMQGTGAVEKVTLLIESGTRF 59
Hs SDCCAG1            MKSRFSTIDLRAVLAELNASLLGMRVNNVYDVDNKTYLIRLQ--KPDFKATLLLESGIRI 58
                      ** *:.:.*:    **.  : * *:.:.*::  :  : :      * .::::.* *

Sp mtr1               HLTKFDR-ENAPLSGFVTKLRKHIKSRRLTGVSQLGTDRVLVFTFGGGANDQDPDWTYYL 117
Sc TAE2               YLTEFSRPIPPTPSGFVVKLRKHLKAKRLTALKQVDQDRILVLQFA--------DGHFYL 112
Dm Caliban            HTTRFEWPKNMAPSGFSMKLRKHLKNKRLEKVQQMGSDRIVDFQFG------TGDAAYHV 113
Hs SDCCAG1            HTTEFEWPKNMMPSSFAMKCRKHLKSRRLVSAKQLGVDRIVDFQFG------SDEAAYHL 112
                      : *.*.      *.*  * ***:* :**  .*:. **:: : *.       :  :::

Sp mtr1               VCEFFAAGNVLLLDGHYKILSLLRVVTFDKDQV----YAVGQKYNLDKNNLVNDNKSQST 173
Sc TAE2               VLEFFSAGNVILLDENRRIMALQRVVLEHENKVGQIYEMFDESLFTTNNESADESIEKNR 172
Dm Caliban            ILELYDRGNVILTDYELTTLYILRPHT-EGENLR---FAMREKYPVER--------AKQP 161
Hs SDCCAG1            IIELYDRGNIVLTDYEYVILNILRFRTDEADDVK---FAVRERYPLDH--------ARAA 161
                      : *::  **::* * .   : : *    . :.:     . :    .         :

Sp mtr1               IPHMTAERLNILLDEISTAYASPTSINEPLPDQQLSSSTKPIKVPKPVSLRKALTIRLGE 233
Sc TAE2               KAEYTSELVNEWIKAVQAKYESDITVIKQLNIQGKEGAKK--KKVKVPSIHKLLLSKVPH 230
Dm Caliban            TKELELEALVKLLENARNGDYLRQILTPNLD----------------------------- 192
Hs SDCCAG1            EPLLTLERLTEIVASAPKGELLKRVLNPLLP----------------------------- 192
                            * :   :           :  *

Sp mtr1               YGNALIEHCLRRSKLDP-----------------------LFPACQLCADETKK------ 264
Sc TAE2               LSSDLLSKNLKVFNIDP-------------------------SESCLNLLEETDS----- 260
Dm Caliban            CGPAVIEHVLLSHGLDNHVIKKETTEETPEAEDKPEKGGKKQRKKQQNTKLEQKPFDMVN 252
Hs SDCCAG1            YGPALIEHCLLENGFS--------------------------GNVKVDEKLETK------ 220
                       .  ::.: *    :.                                  .

Sp mtr1               --NDLLAAFQEADSILAAVNKPPVKGYIFSLEQALTNAADPQHPEECTTLYEDFHPFQPL 322
Sc TAE2               -LAELLNSTQLEYNQLLTTTD--RKGYILAKR--NENYISEKDTADLEFIYDTFHPFKPY 315
Dm Caliban            DLPILQQAVKDAQELIAEGNSGKSKGYIIQVK-EEKP--TENGTVEFFFRNIEFHPYLFI 309
Hs SDCCAG1            DIEKVLVSLQKAEDYMKTTSNFSGKGYIIQKR-EIKPSLEADKPVEDILTYEEFHPFLFS 279
                          :  . :   . :  ..   ****:  .        . . :      ***:

Sp mtr1               QLVQANRK--CMEFPT-YNECVDEFFSSIEAQKLKKRAHDRLATAERRLESAKEDQARKL 379
Sc TAE2               INGGDTDSSCIIEVEGPYNRTLDKFFSTIESSKYALRIQNQESQAQKKIDDARAENDRKI 375
Dm Caliban            QFKNFE----KATFES-FMEAVDEFYSTQESQKIDMKTLQQEREALKKLSNVKNDHAKRL 364
Hs SDCCAG1            QHSQCP----YIEFES-FDKAVDEFYSKIEGQKIDLKALQQEKQALKKLDNVRKDHENRL 334
                                  .   : . :*:*:*.*..*   :  ::  * :::...: :: .::

Sp mtr1               QSLQDAQATCALRAQAIEMNPELVEAIISYINSLLNQGMDWLDIEKLIQSQKRRSPVAAA 439
SC TAE2               QALLDVQELNERKGHLIIENAPLIEEVKLAVQGLIDQQMDWNTIEKLIKSEQKKGNRIAQ 435
Dm Caliban            EELTKVQDVDRKKAELITSNQSLVDNAIRAVQSAIASQLSWPDIHELVKEAQANGDAVAS 424
Hs SDCCAG1            EALQQAQEIDKLKGELIEMNLQIVDRAIQVVRSALANQIDWTEIGLIVKEAQAQGDPVAS 394
                      : * ..*    :.. *  * :::     :.. : . :.* *  :::. : ..   *

Sp mtr1               IQ-IPLKLIKNAVTVFLPNPE---SVDNSDES----------SETSDDDLDDSDDDNKVK 485
Sc TAE2               LLNLPLNLKQNKISVKLDLSSKELNTSSDEDNESEGNTTDSSSDSDSEDMESSKERSTKS 495
Dm Caliban            SI-KQLKLETNHISLMLSDPY---DNDEDDD----------------------------L 452
Hs SDCCAG1            AI-KELKLQTNHVTMLLRNPY---LLSEEEDDDVDGDVNVEKNETEPPKGKKKKQKNKQL 450
                           *:*  * ::: *  .      ...::

Sp mtr1               EGKVSSKFIAVELDLSLGAFANARKQYELRREALIKETKTAEAASKALKSTQRKIEQDLK 545
Sc TAE2               MKRKSNEKINVTIDLGLSAYANATEYFNIKKTSAQKQKKVEKNVGKAMKNIEVKIDQQLK 555
Dm Caliban            KDPEVT---VVDVDLALSAWANARRYYDMKRSAAQKEKKTVDASQKALKSAERKTQQTLK 509
Hs SDCCAG1            QKPQKNKPLLVDVDLSLSAYANAKKYYDHKRYAAKKTQKTVEAAEKAFKSAEKKTKQTLK 510
                           .    * :**.*.*:*** . :: :: :  *  :. .   **:*. : * .* **

Sp mtr1               RSTTADTQRILLGRKTFFFEKFHWFISSEGYLVLGGRDAQQNELLFQKYCNTGDIFVCAD 605
Sc TAE2               KKLKDSHSVLKKIRTPYFFEKYSWFISSEGFLVMMGKSPAETDQIYSKYIEDDDIYMSNS 615
Dm Caliban            EVRTISN--IVKARKVFWFEKFYWFISSENYLVIGGRDAQQNELIVKRYMRPKDIYVHAE 567
Hs SDCCAG1            EVQTVTS--IQKARKVYWFEKFLWFISSENYLIIGGRDQQQNEIIVKRYLTPGDIYVHAD 568
                      .  .     :   *. ::***: ******.:*:: *:.  :.: : .:*    **::  .
```

Continued

Fig. S1. Sequence alignment comparison of *S. pombe* mtr1p. CLUSTAL 2.1 multiple sequence alignment.

Biology Open (2014) 000, 1–6 doi:10.1242/bio.20148607

Fig. S1. continued

```
Sp mtr1               LPKSSIIIVKNKNPHDPIPPNTLQQAGSLALASSKAWDSKTVISAWWVRIDEVSKLAPTG 665
Sc TAE2                FN--SHVWIKN-PEKTEVPPNTLMQAGILCMSSSEAWSKKISSSPWWCFAKNVSKFDGSD 672
Dm Caliban            IQGASSVIIQN-PTGEEIPPKTLLEAGSMAISYSVAWDAKVVTNSYWVTSDQVSKTAPTG 626
Hs SDCCAG1            LHGATSCVIKN-PTGEPIPPRTLTEAGTMALCYSAAWDARVITSAWWVYHHQVSKTAPTG 627
                      :   :   ::*      :**.** :** :.:. * **. :   ..:*   .:***   :.

Sp mtr1               -EILPTGSFAIRAK--KNYLPPTVLIMGYGILWQLDE----KSSERRKARRLEMEVVETQ 718
Sc TAE2                NSILPEGAFRLKNENDQNHLPPAQLVMGFGFLWKVKTSGNEDNGDDDEEEEEEEEEEEEE 732
Dm Caliban            -EYLATGSFMIRGK--KNFLPSCHLTMGLSLLFKLEDSFIERHLGERKVRSLEDDQIDPN 683
Hs SDCCAG1            -EYLTTGSFMIRGK--KNFLPPSYLMMGFSFLFKVDESCVWRHQGERKVRVQDEDMETLA 684
                       . *. *:* :: :  :*.**.  * ** .:*:::.           : .  : :

Sp mtr1               GKVSELKMEGTSVTSEDNIQDVVSEVSYNEDTNNQSTPDTTGSDIHIVS----------- 767
Sc TAE2                EEEEEEEEEEKEEEEKEEEQQQDEDDSNEVNG---------------------------- 765
Dm Caliban            VKENEVEHDLLSDNEDADSNINLSEPSSNTEITAFPNTEVKIEH---------------- 727
Hs SDCCAG1            SCTSELISEEMEQLDGGDTSSDEDKEEHETPVEVELMTQVDQEDITLQSGRDELNEELIQ 744
                         .*   :  .  .  : .    :

Sp mtr1               ---------EKRGKKGSKVITAKKVSAKER----------REARRARRQTALEESLKAPI 808
Sc TAE2                ------------LEKGGDSNDSTK-----------------NNSFEHDNLEKDIEKHCTI 796
Dm Caliban            --------DTGRIIVRSDSVNPEIEETKE-----------SEVVLDKILKKTDDEETTII 768
Hs SDCCAG1            EESSEDEGEYEEVRKDQDSVGEMKDEGEETLNYPDTTIDLSHLQPQRSIQKLASKEESSN 804
                                       .                       .    :

Sp mtr1               SIEDATDPQTILAILKQK-------------------KAKKKHAAREMEISSQIPSN--- 846
Sc TAE2                SSDTDSDSGNAKAKNDNS-----------------STQRILDEPGVPISLIENINSN--- 836
Dm Caliban            LAGPSR--KKQVSAKKTK-----------------EDKARAKQEAAKQEVPPVSSEPKNP 809
Hs SDCCAG1            SSDSKSQSRRHLSAKERREMKKKKLPSDSGDLEALEGKDKEKESTVHIETHQNTSKNVAA 864
                                  :  .                     :    .     .      .

Sp mtr1               ------DSSNVQTPTAESEIEEDGVSEPISAEVIEDQSRNSEAENEKGLSTEQRDEKK-- 898
Sc TAE2                ----VRGKRGKLKKIQKKYADQDETERLLRLEALGTLKGIEKQQQRKKEEIMKREVREDR 892
Dm Caliban            SQ-VKRGQKGKLKKMKQKYKDQDDEEREIRMMILKSSG--KEKPQASADKVVEKSESTKE 866
Hs SDCCAG1            VQPMKRGQKSKMKKMKEKYKDQDEEDRELIMKLLGSAGSNKEEKGKKGKKGKTKDEPVKK 924
                            .. .  .   :.  ::*  .. :    :      .:    .  .   :.

Sp mtr1               --------------------------------HAKVESFQRQEMPRSLFEEIFFAIDSLT 926
Sc TAE2                KNKREKQR------------------------RLQALKFTKKEKARVNYDKHK---SELK 925
Dm Caliban            YVKPEKS-------------------------AAPKNPVELDDADEVPVGGDVDVLNSLT 901
Hs SDCCAG1            QPQKPRGGQRVSDNIKKETPFLEVITHELQDFAVDDPHDDKEEQDLDQQGNEENLFDSLT 984
                                                               .:             ..*.

Sp mtr1               PNPQQQDTVINAVPTFAPYNAMTKFNQKVKVMPGTGKVGKAARESIAYFMKKLPKSS--- 983
Sc TAE2                PSLDKGDVVDDIIPVFAPWPALLKYKYKVKIQPGSAKKTKTLTEILHYFKSRPLDGSSTD 985
Dm Caliban            GQPHEGDELLFAIPVVAPYQALQNYKFKVKLTPGTGKRGKAAKLALNIFAKEKSCSA--- 958
Hs SDCCAG1            GQPHPEDVLLFAIPICAPYTTMTNYKYKVKLTPGVQKKGKAAKTALNSFMHSKEATA--- 1041
                       . .  * :   :*  **:  : ::: ***: **  *  *:    :  *       :

Sp mtr1               ------KEAAYLENLKDGEIVAPISVSRLKMVFGSSGNTKKSKK--------- 1021
Sc TAE2                NEMDWPQEHEMIKGLKEQDLVLLLCVDKLKVTIAGQKSTKNGGNSSKKGKKKR 1038
Dm Caliban            ------REKDLLKSIKEESLARNIPG-KVKLSAPQLQKYHK------------ 992
Hs SDCCAG1            ------REKDLFRSVKDTDLSRNIPG-KVKVSAPNLLNVKRK----------- 1076
                            :*   :..:*: .:   :   ::*:        :.
```

* = 100% aligned

: = one residue minimum not aligned, but charge of the residue is conserved

. = minimum of 50% conserved

Table S1. List of strains

Strain	Genotype	Source	Figure
TP 449	GFP-atb2:NatR ade6-M210 leu1-32 ura4-D18 h+	M. Sato	Fig. 1, Fig. 3A
TP 1004	mtr1Δ::KanR GFP-atb2:NatR ade6-M210 leu1-32 ura4-D18 h−	This study	Fig. 1, Fig. 4B
TP 1026	mtr1-GFP:KanR mcherry-atb2:HphR ade6-M210? leu1-32 ura4-D18 leu1-32 h+	This study	Fig. 2
TP 1096	mtr1Δ::KanR Pnmt1-mtr1-YFP:LEU1 mcherry-atb2:HphR h+	This study	Fig. 2
TP 1067	mtr1Δ::KanR mcherry-atb2:HphR h−	This study	Fig. 2
TP 1250	mal3-GFP:KanR ade6-M216 leu1-32 ura4-D18 h+	This study	Fig. 3G
TP 1438	mtr1Δ::NatR mal3-GFP:KanR ade6-M216 leu1-32 ura4-D18 h+	This study	Fig. 3G
TP 1063	mal3Δ::HIS3 GFP-atb2:NatR ade6-M210 leu1-32 ura4-D18 his3 h−	This study	Fig. 3A–E
TP 1065	mtr1Δ::KanR mal3Δ::HIS7 GFP-atb2:NatR h−	This study	Fig. 3A–E
TP 319	mCherry-atb2:HphR ade6-M210? leu1-32 ura4-D18 h+	This study	Fig. 2B, Fig. 4B
TP 1406	rps1801Δ::KanR GFP-atb2:NatR ade6-M210 leu1-32 ura4-D18 h+	This study	Fig. 4
TP 1863	rps1801Δ::KanR mal3Δ::HIS3 GFP-atb2:NatR ade6-M210 leu1-32 ura4-D18 h+	This study	Fig. 4
